# Applicability, safety, and biological activity of regulatory T cell therapy in liver transplantation

**DOI:** 10.1111/ajt.15700

**Published:** 2020-02-03

**Authors:** Alberto Sánchez‐Fueyo, Gavin Whitehouse, Nathali Grageda, Matthew E. Cramp, Tiong Y. Lim, Marco Romano, Sarah Thirkell, Katie Lowe, Laura Fry, Julie Heward, Alex Kerr, Jakia Ali, Chris Fisher, Gillian Lewis, Andrew Hope, Elisavet Kodela, Mike Lyne, Farzin Farzaneh, Shahram Kordasti, Irene Rebollo‐Mesa, Juan Jose Lozano, Niloufar Safinia, Nigel Heaton, Robert Lechler, Marc Martínez‐Llordella, Giovanna Lombardi

**Affiliations:** ^1^ Institute of Liver Studies MRC Centre for Transplantation Department of Inflammation Biology Faculty of Life Sciences & Medicine King's College London London UK; ^2^ MRC Centre for Transplantation Peter Gorer Department of Immunobiology Faculty of Life Sciences & Medicine King's College London London UK; ^3^ Hepatology Research Group Plymouth University Peninsula Schools of Medicine and Dentistry Southwest Liver Unit Derriford Hospital Plymouth Hospitals NHS Trust Plymouth UK; ^4^ NIHR Biomedical Research Centre Guy's and St Thomas' NHS Foundation Trust and King's College London London UK; ^5^ School of Cancer and Pharmaceutical Sciences King's College London London UK; ^6^ Systems Cancer Immunology Lab Comprehensive Cancer Centre King’s College London, & Haematology Department Guy’s Hospital London UK; ^7^ Biostatistics Institute of Psychiatry, Psychology and Neuroscience King's College London London UK; ^8^ Bioinformatic Platform Biomedical Research Center in Hepatic and Digestive Diseases (CIBEREHD) Instituto de Salud Carlos III Spain

**Keywords:** cellular transplantation (nonislet), immunosuppression/immune modulation, liver transplantation/hepatology, T cell biology, tolerance, translational research/science

## Abstract

Regulatory T cells (Tregs) are a lymphocyte subset with intrinsic immunosuppressive properties that can be expanded in large numbers ex vivo and have been shown to prevent allograft rejection and promote tolerance in animal models. To investigate the safety, applicability, and biological activity of autologous Treg adoptive transfer in humans, we conducted an open‐label, dose‐escalation, Phase I clinical trial in liver transplantation. Patients were enrolled while awaiting liver transplantation or 6‐12 months posttransplant. Circulating Tregs were isolated from blood or leukapheresis, expanded under good manufacturing practices (GMP) conditions, and administered intravenously at either 0.5‐1 million Tregs/kg or 3‐4.5 million Tregs/kg. The primary endpoint was the rate of dose‐ limiting toxicities occurring within 4 weeks of infusion. The applicability of the clinical protocol was poor unless patient recruitment was deferred until 6‐12 months posttransplant. Thus, only 3 of the 17 patients who consented while awaiting liver transplantation were dosed. In contrast, all six patients who consented 6‐12 months posttransplant received the cell infusion. Treg transfer was safe, transiently increased the pool of circulating Tregs and reduced anti‐donor T cell responses. Our study opens the door to employing Treg immunotherapy to facilitate the reduction or complete discontinuation of immunosuppression following liver transplantation.

## Abstract 2

CD4+Foxp3+ regulatory T cells isolated from either patients awaiting liver transplantation or recently transplanted recipients can be expanded in large numbers ex vivo and safely infused into recipients on immunosuppression.

**Abbreviations**
CAR: chimeric antigen receptor
CCL: C‐C motif chemokine ligand
CCR: chemokine receptor
CD: cluster of differentiation
CMV: cytomegalovirus
CTCAE: National Cancer Institute's Common Terminology Criteria for Adverse Events
CTLA4: cytotoxic T lymphocyte‐associated protein 4
CXC: C‐X‐C motif chemokine
CXCR: C‐X‐C chemokine receptor
CyTOF: Time of Flight Mass Cytometry
EBV: Epstein Barr virus
FDA: Food and Drug Administration
FOXP3: Forkhead Box P3
GATA3: Gata binding protein 3
GMP: good manufacturing practices
MELD: Model for End‐Stage Liver Disease
PBMC: peripheral blood mononuclear cell
PD1: programmed cell death protein 1
Tregs: CD4^+^ Foxp3^+^ regulatory T cells

## 1 INTRODUCTION

Regulatory T cells (Tregs) are a subset of cluster of differentiation (CD)4‐positive T cells that constitutively express the Forkhead Box P3 (Foxp3) transcription factor and have the capacity to migrate to sites of inflammation and exert a wide range of immunosuppressive effects. Animal studies indicate that Tregs play a key role in maintaining immune homeostasis and preventing autoimmunity.^1^ Furthermore, they can recognize allogeneic major histocompatibility complex (MHC) molecules and suppress allograft rejection, and are essential for the induction and maintenance of transplantation tolerance through the mechanisms of “linked suppression” and “infectious tolerance.”^2^

Although human Tregs constitute a small proportion (5%‐7%) of circulating CD4^+^ T cells, they are attractive candidates for immunotherapeutic purposes given that they can be isolated and expanded in large numbers in vitro without losing their immunoregulatory properties.^3^ Clinical studies have demonstrated the safety of Treg adoptive transfer in graft‐versus‐host disease and type 1 diabetes mellitus.^4^, ^5^, ^6^, ^7^ Furthermore, a number of trials have been initiated both in kidney and in liver transplantation.^8^, ^9^ Liver transplantation constitutes an appealing clinical setting to evaluate the effects of Treg transfer given the lower immunogenicity of liver allografts and the substantial clinical experience that has been derived from trials of complete immunosuppression discontinuation.^10^ In this setting, infusion of a single dose of a Treg‐enriched autologous leukocyte cell product (generated by culturing peripheral blood mononuclear cells (PBMCs) with irradiated donor leukocytes in the presence of co‐stimulation blockade), was recently shown to successfully induce operational tolerance in 7 of 10 splenectomized living donor liver transplant recipients treated with cyclophosphamide and conventional immunosuppression.^11^

Despite these encouraging early results, key questions regarding the overall clinical applicability of Treg immunotherapy, the optimal clinical design, and the immunological effects of Treg infusion in human liver transplant recipients remain to be answered. We recently described the first good manufacturing practices (GMP)–compliant protocol for the ex vivo expansion of polyclonal Tregs from prospective liver transplant recipients.^12^ This protocol, which included up to three rounds of stimulation in the presence of rapamycin, was successful in expanding circulating Tregs >100‐fold, maintained their Foxp3 expression levels, and increasing their suppressive function. It is important to note that expanded Tregs exhibited a stable noninflammatory phenotype even after being challenged with a cocktail of inflammatory cytokines. We report here the results of a First‐in‐Human Phase I clinical trial evaluating the safety and immunological effects of purified, ex vivo expanded and adoptively transferred autologous polyclonal Tregs in adult liver transplant recipients.

## 2 MATERIALS AND METHODS

### 2.1 Study design

This was a two‐site, open‐label, dose escalation, Phase I clinical trial conducted at King's College Hospital London and University Hospitals Plymouth (UK), assessing the safety, applicability, and biological activity of autologous Treg immunotherapy in the setting of adult cadaveric liver transplantation. Participants received a single intravenous infusion of ex vivo expanded autologous polyclonal Tregs 3‐16 months after liver transplantation. The trial was approved by the UK National Research Ethics Service (Reference 13/SC/0604, 10/1/2014) and the Medicines and Healthcare Products Regulatory Agency (MHRA), and was registered at ClinicalTrials.gov (identifier NCT02166177). All data supporting the results in this article will be archived in an appropriate public repository.

### 2.2 Participants

Patients were initially enrolled while awaiting liver transplantation and their participation was confirmed on the day of transplantation. Inclusion criteria at the time of transplantation were the following: (1) age 18‐70 years; (2) Model for End‐Stage Liver Disease (MELD) score ≤25; (3) no previous transplantation or need for simultaneous liver‐kidney transplantation; (4) absence of autoimmune disease, active viral disease, Epstein‐Barr virus seronegativity or hepatocellular carcinoma outside of Milan criteria; (5) leukocyte count >1500/µL and platelet count >50 000 µL; (6) recipient of a brain‐dead liver donor; (7) recipient of a cardiac death liver donor if donor age <50‐years‐old, warm ischemia time <20 minutes, and cold ischemia time <8 hours. For Treg isolation, 250 mL of whole blood was collected during the induction of anesthesia. Participants received thymoglobulin induction (three doses of 1.5 mg/kg, i.v., between posttransplant days 1 and 7), tacrolimus (1 mg twice daily on posttransplant day 1 with doses subsequently adjusted to reach 5‐8 ng/mL trough levels), and methylprednisolone (500 mg intraoperatively followed by tapering and discontinuation on posttransplant week 10). Between posttransplant weeks 6 and 8, rapamycin (5‐8 ng/mL trough levels) was initiated and levels of tacrolimus (2‐5 ng/mL) were decreased. Three months after transplant a liver biopsy was performed to exclude subclinical allograft damage, and patients were admitted for Treg infusion.

Due to the difficulties of enrolling patients before transplantation when following the protocol described, 26 months after its initiation the trial design was amended and all subsequent patients were recruited 6‐12 months after transplant. Otherwise, the same inclusion/exclusion criteria were maintained. Immediately after enrollment, patients had their immunosuppressive regimen switched to combined tacrolimus and rapamycin (trough levels 2‐5 ng/mL and 2‐8 ng/mL, respectively), and 2 months afterward they underwent leukapheresis to collect the starting material for Treg manufacture. This was followed by a protocol liver biopsy and by the infusion of Tregs 4 months after enrollment. The amended study protocol did not require thymoglobulin induction. This Phase I trial did not include attempts at immunosuppression discontinuation.

### 2.3 Dose escalation

Two doses of expanded Tregs were assessed: 0.5‐1 million Tregs/kg and 3‐4.5 million Tregs/kg. Dose escalation criteria were as follows: (1) after the treatment of the first three patients with 0.5‐1 million Tregs/kg, if dose‐limiting toxicities were observed in one of three patients, the cohort would be expanded to three additional patients at the same dose; (2) if toxicity was observed in two or more of the six patients, dose escalation would stop; (3) if zero of three or one or fewer of size dose‐limiting toxicities were observed in the three or six patients, then the dose would be defined as well tolerated and a new cohort of three to six patients would be treated with 3‐4.5 million Tregs/kg.

### 2.4 Study endpoints

The primary endpoint was the rate of dose‐limiting toxicities within the 4 weeks following infusion. Dose‐limiting toxicities were defined as: (1) occurring in the first 72 hours postinfusion, including: National Cancer Institute's Common Terminology Criteria for Adverse Events (CTCAE; Version 4.0) grade 2 or higher cytokine release syndrome, grade 2 or higher injection site reaction, grade 2 or higher fever and/or rigors, grade 3 or higher bronchospasm, grade 3 or higher hypoxia; (2) occurring in the first 30 days of the infusion including: grade 3 or higher infection, grade 3 or higher hematological complication, any CTCAE grade 3 or higher toxicity not clearly related to underlying disease, moderate or severe acute rejection. Secondary endpoints were: acute and chronic toxicity associated with Treg infusion; incidence of major/opportunistic infections; malignancy; rejection; graft loss; patient mortality; sequential liver and renal function tests; immunosuppressive drug doses and levels; and changes in immunological parameters following Treg infusion.

### 2.5 Isolation and manufacture of polyclonal Tregs

Two hundred fifty milliliters of whole blood or 180 mL of leukapheresis product was collected and transferred to the GMP Cell Therapy. The manufacture protocol as well as the phenotypic characteristics, functional properties, and stability of the expanded Tregs have been reported previously^12^ and are described in detail as Supplementary Information.

### 2.6 Treg infusion

Patients were admitted on the day of infusion. The cryopreserved Treg product was thawed in a 37°C water bath, diluted in an infusion bag containing 50 mL of 5% human albumin solution (Albunorm, Octapharma), and infused via a peripheral cannula over 15 minutes with an additional 50 mL of 5% human albumin added to the bag to ensure delivery of the full dose. Premedication consisted of oral paracetamol (1 g) and chlorphenamine (4 mg) 30 minutes prior to infusion. All patients were monitored for 12 hours postinfusion prior to discharge.

### 2.7 Immunomonitoring studies

***Specimens***: Sequential peripheral blood samples were collected at nine different time points between enrollment and the end of follow‐up. Blood was collected into EDTA vacutainers and employed fresh in flow cytometric experiments or used to isolate PBMCs that were cryopreserved. Serum specimens were isolated and cryopreserved.

***Quantification of donor‐specific alloimmune responses***: The proportion of alloreactive CD8^+^ memory T cells was assessed in cryopreserved PBMCs collected immediately before Treg infusion, and 7 days and 1 month afterward, employing the U.S. Food and Drug Administration (FDA)–approved Pleximmune test.^13^, ^14^ This assay uses flow cytometry to quantify the number of recipient CD8^+^ CD45RO^+^ memory T cells expressing CD154 following 16 hours of culture with surrogate donor PBMCs (matched to donor at a minimum of one antigen each at human leukocyte antigen [HLA]‐A, ‐B, and ‐DR loci or 6‐loci mismatched third‐party PBMCs). Similar experiments were conducted to quantify the frequency of CD154‐positive memory CD8^+^ T cells following culture with an overlapping peptide mix of CMV pp65 antigen.

***Detection of serum cytokines and chemokines***: We employed the LEGENDplex Multi‐Analyte Flow Assay kits (Human Cytokine panel 2, Human Proinflammatory Chemokine panel, and Human Th Cytokine panel) according to the manufacturer's instructions.

***Flow cytometry and time‐of‐flight mass cytometry (CyTOF) immunophenotyping***: The flow cytometry reagents and staining protocols employed were designed and standardized in collaboration with the ONE Study EU Consortium and have already been described (Table S1).^15^ The antibody panel, staining protocol, and data analysis strategy for the CyTOF 16, 17, 18, 19, 20experiments are described as Supplementary Information.

## 3 RESULTS

### 3.1 Patient flow, changes to study design, and clinical outcomes

**Figure 1:**
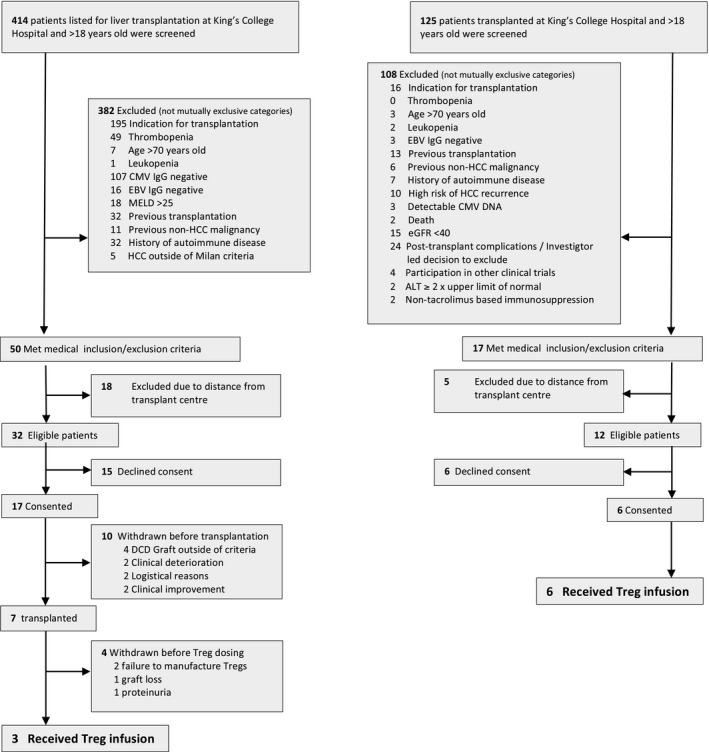
Identification, enrollment, and follow‐up of eligible subjects. A, Original trial design targeting patients awaiting liver transplantation. B, Amended trial design targeting patients 6‐12 months posttransplant

Between 2/6/2014 and 9/8/2016, a total of 414 patients awaiting liver transplantation were screened to participate in the trial; 17 were consented, 10 withdrew from the study before or at the time of transplantation, and 7 were transplanted (Figure 1). Of seven patients who were transplanted, four were withdrawn from the study before Treg infusion due to: (1) hepatic artery occlusion requiring retransplantation followed by death caused by disseminated fungal infection; (2) development of proteinuria that precluded initiation of rapamycin; and (3) failure to manufacture the required number of Tregs (in two patients). The remaining three patients received an infusion of 1 million Tregs/kg 83‐110 days posttransplant and were followed for a total of 12 months. Following the protocol amendment, we screened 125 patients between 12/9/2016 and 1/3/2017 and consented 6 patients 221‐354 days after transplant. All six proceeded to receive an infusion of 4.5 million Tregs/kg (Figure 1) 112‐151 days after enrollment and were followed for 6 months following Treg infusion. All nine patients received the stipulated immunosuppression regimen as per protocol; there were no episodes of rejection during the follow‐up period and all protocol liver biopsies performed before Treg infusion revealed normal histology or minimal changes (data not shown). Patient characteristics are summarized in Tables 1 and S5.

**Table 1:** Clinical and demographic characteristics of enrolled patients

ID	Dose cohort (10^6^ Tregs/kg)	Sex	Age	Liver disease etiology	Graft type & donor age (y)	Time of Treg infusion (posttransplant days)	Lymphocyte count at time of Treg infusion (10^9^/L)	Bilirubin at time of infusion (μmol/L)	AST at time of infusion (IU/L)	Creatinine at time of infusion (μmol/L)	Lymphocyte count at 6 mo (10^9^/L)	Bilirubin at 6 mo (μmol/L)	AST at 6 mo (IU/L)	Creatinine at 6 mo (μmol/L)
P01	1.0	M	42	Cryptogenic cirrhosis	DBD 70	83	0.61	7	22	56	1.03	16	26	72
P02	1.0	M	57	Haemochromatosis	DBD 74	110	0.41	6	21	148	0.63	10	36	145
P03	1.0	M	67	Alcoholic cirrhosis	DBD 76	95	0.41	4	11	133	NA	NA	NA	NA
P04	4.5	F	47	SALF	DBD 39	335	2.20	6	28	74	2.26	5	36	76
P05	4.5	M	57	HCC + HCV cirrhosis	DBD 24	413	1.48	12	26	86	1.02	10	33	88
P06	4.5	M	48	Alcoholic cirrhosis	DBD 62	481	1.21	11	26	65	1.50	8	22	58
P07	4.5	M	46	HCC + HCV cirrhosis	DBD 75	406	1.41	12	26	60	1.32	10	26	72
P08	4.5	M	63	Alcoholic cirrhosis	DBD 47	446	1.34	7	22	85	1.17	6	30	85
P09	4.5	M	58	Alcoholic cirrhosis	DCD 61	438	0.71	5	20	100	1.08	5	19	92

Abbreviations: Treg, CD4^+^ CD25^+^ Foxp3^+^ regulatory T cell; SVR, sustained viral response; DBD, donation after brain death; DCD, donation after circulatory death; HCV, hepatitis C virus; HCC, hepatocellular carcinoma; ID, study number; SALF, seronegative subacute liver failure; NA, not available.

### 3.2 Manufacture of ex vivo expanded Tregs

Tregs were isolated from 11 patients (5 from whole blood and 6 from leukapheresis). The manufacture process failed in two patients (all of them from the first cohort of patients). The first case was due to an insufficient number of Tregs (49 million Tregs), likely resulting from the very low number of Tregs isolated from blood (1.5 million Tregs as compared to 5.9 million, which was the mean from all whole blood Treg isolations). The second failure was due to a low frequency of Tregs in the final product (46% of CD4^+^ CD25^+^ Foxp3^+^). In the nine successful manufacture runs, cells were expanded 21‐ to 486‐fold, yielding between 1250 and 22 530 million cells containing 61%‐92% Tregs. As compared to whole blood, the use of leukapheresis products allowed a reduction in the duration of Treg culture (from 36 to 24 days) and the need for lower expansion rates to achieve the target dose (Table 2). The use of immunosuppressive drugs by the trial participants at the time of leukapheresis did not hamper the Treg manufacture process, as Tregs were successfully expanded from all six recipients recruited 6‐12 months after transplant (Table 2).

**Table 2:** Characteristics of the Treg manufactured product

ID	Blood/leukopheresis volume (L)	Starting Treg number (×10^6^)	Final Treg number (×10^6^)	Expansion (fold‐change)	% Viability^a^	% CD4^+^ CD25^+^ FoxP3^+^ a	% CD8^+^ a	1:1 Suppression^a^ (%)	1:5 Suppression^a^ (%)	1:10 Suppression^a^ (%)	Total number Tregs infused (×10^6^)	Viability after thawing (%)
P01	0.248	7.2	3480	486	96.8	85.3	1.4	94.18	83	65.32	96	77.4
P02	0.291	3.2	1250	390.6	95.1	69.9	0.4	93.2	95.4	92.2	65	64.5
P03	0.280	8.8	3193	362.9	95.7	66.5	2.6	72.596	76.9	16.667	88	58
P04	0.175	105	4089	38.9	97	78	2.3	97.9	90.9	88.1	468	77.9
P05	0.168	190	22 530	118.6	96.5	77.4	0.3	97.36	97.58	81.82	395	73.1
P06	0.171	136.3	9145	67.1	98.6	82.8	3.2	86.15	95.96	92.09	440	76.7
P07	0.166	253.3	7893	31.2	98.9	83.8	0.1	95.9	79.7	86.2	339	89
P08	0.171	288.5	5986	20.8	97.6	61.2	0.2	99.1	98.6	90.7	375	84.9
P09	0.163	102	4702	46.1	96.1	91.8	0.3	97.3	96.2	72.3	340	78

a Assessed before Treg product cryopreservation.

### 3.3 Characteristics of manufactured Tregs and effects on the phenotype of circulating immune cells following infusion

**Figure 2:**
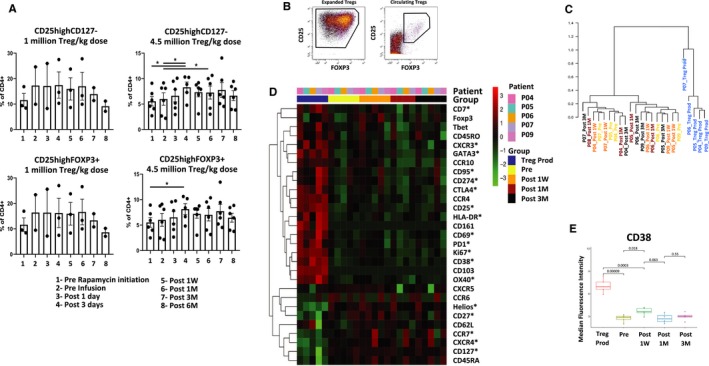
In‐depth phenotypic characterization of expanded and circulating Tregs employing CyTOF. A, Barplots displaying the results of flow cytometric experiments assessing the sequential changes in the proportion of Tregs (defined by either CD4^+^CD25highCD127^−^ or CD25highFOXP^+^ expression) among circulating CD4^+^ T cells in the three patients receiving 1 million Tregs/kg and the six patients receiving 4.5 million Tregs/kg. Asterisks denote *P* < .05. B, Representative dot plot showing the expression of CD25 and Foxp3 in expanded (left panel) and circulating (right panel) Tregs after gating for CD3^+^, CD4^+^, and CD8^−^ cells employing CyTOF. C, Dendogram derived from a hierarchical clustering analysis of the patterns of variation in the expression of the 29 phenotypic markers shown in D in the 24 samples analyzed using CyTOF. The horizontal axis corresponds to the samples; the vertical axis corresponds to the dissimilarity between clusters. D, Heatmap displaying the median expression of 29 markers employed to characterize expanded and circulating Tregs by CyTOF (gated as described in B) before and at different time points following cell infusion: Preinfusion, 1‐week postinfusion (Post 1W), 1‐month postinfusion (Post 1M), 3‐months postinfusion (Post 3M). Rows represent individual markers and columns represent patient samples. The color in each cell reflects the relative expression level of the corresponding marker in the corresponding sample. Asterisks denote *P* < .05 when comparing the expanded Tregs and the circulating preinfusion Tregs. E, Expression levels of CD38 in expanded and circulating Tregs at different time points following cell infusion [Color figure can be viewed at https://www.wileyonlinelibrary.com]

**Figure 3:**
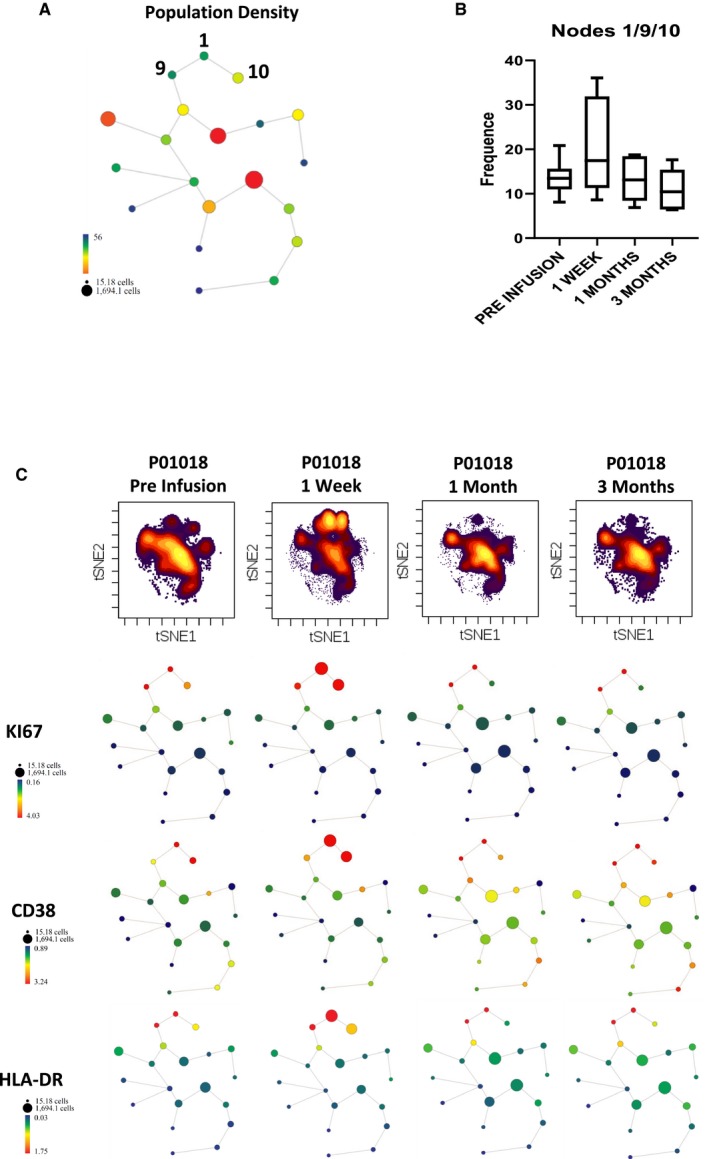
Sequential changes in circulating Treg subsets following infusion of 4.5 million Tregs/kg. A, SPADE algorithm clustering of circulating Tregs before cell infusion based on the viSNE analysis of the markers assessed by CyTOF and described in Figure 2B. Data show all viable single cells hierarchically clustered according to similar protein expression levels. The nodes 1, 9, and 10 identify the Treg subpopulations expressing the highest levels of the 10 parameters more differentially expressed in expanded Tregs as compared to preinfusion circulating Tregs (CD38, Ki67, OX40, CD25, CD69, GATA3, CCR4, CTLA4, PD1, and HLA‐DR). Bubble size and color intensity correspond to population density. B, Cumulative frequency of circulating Tregs clustered on nodes 1, 9, and 10 at different time points before and after cell infusion grouping all six patients together. C, Representative viSNE density plots (top panel) and SPADE analyses (bottom panel) corresponding to circulating Tregs assessed at different time points before and after infusion. For SPADE analyses, bubble size represents population density and color denotes the magnitude of expression of the three representative markers (KI67, CD38, and HLA‐DR). An increase in nodes 1, 9, and 10 is noticeable 1 week, but not 1 month or 3 months, after cell infusion [Color figure can be viewed at https://www.wileyonlinelibrary.com]

In the six patients who received the 4.5 million Tregs/kg infusion, a transient increase in circulating Tregs was noticeable by flow cytometry as soon as 3 days after infusion and persisted for 1 month. This increase was larger than what was observed after initiating rapamycin and was not detected in the three patients receiving 1 million Treg/kg (Figure 2A). To better understand the fate of the infused Tregs and their impact on the preexisting Treg compartment in the six patients receiving 4.5 million Tregs/kg, we conducted an in‐depth phenotypic characterization using CyTOF in sequentially collected samples (Figure 2B‐E). We first performed a hierarchical clustering analysis to compare the phenotypic heterogeneity of expanded and circulating Tregs. The expanded Tregs were more homogeneous than the corresponding circulating Tregs, reflecting the effects of the prolonged in vitro culture (Figure 2C). A more detailed analysis revealed that the expanded Tregs were more proliferative than the preinfusion circulating Tregs (as assessed by Ki67 expression) and exhibited higher levels of CD25, CTLA4, CD38, Gata binding protein 3 (GATA3), programmed cell death protein 1 (PD1), CD274 (PD Ligand 1), OX40, CD69, HLA‐DR, CD7, and lower levels of Helios, chemokine receptor (CCR)‐7, C‐X‐C chemokine receptor (CXCR)‐4, and CD127 (Figure 2D). We next investigated whether, following infusion, circulating Tregs exhibited changes in the markers that were most characteristic of the manufactured cells. One week after infusion we detected a significant increase in the expression of CD38, which was no longer detected 3 weeks later (Figure 2E). A similar trend was observed for Ki67, CD7, HLA‐DR, CD274, PD1, and CTLA4 (Figure S1). To better track the infused Tregs and to explore their impact on the population structure of the pool of preexisting circulating Tregs, we identified the three subpopulations of circulating Tregs that most resembled expanded Tregs phenotypically (Figure 3A and S2) and plotted their evolution over time. In keeping with the flow cytometric experiments, the density of the three subpopulations increased noticeably 7 days after infusion, but this was no longer apparent 1‐month postinfusion (Figure 3B,C). We performed exhaustive immunophenotypic experiments on circulating non‐Treg immune cell subsets as well, using both flow cytometry (Tables S3 and S4) and CyTOF (Figures S3 and S4), but observed no significant changes in association with the infusion of Tregs.

### 3.4 Safety of Treg infusion

**Figure 4:**
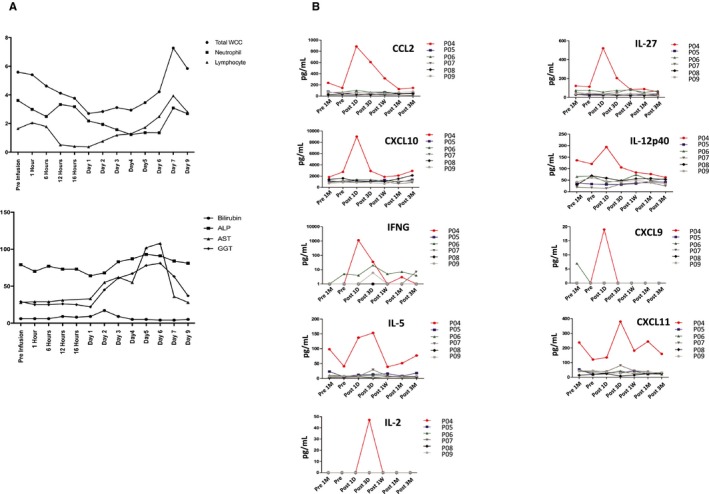
Liver tests and serum cytokine patterns in patients receiving Treg infusion. A, Sequential changes in liver tests and blood cell count of P04 following infusion of 4.5 million Treg/kg. B, Sequential changes in serum cytokine levels in all six patients receiving 4.5 million Tregs/kg [Color figure can be viewed at https://www.wileyonlinelibrary.com]

No adverse events were observed after infusing 1 million Tregs/kg in Patients P01‐P03. Patient P04, however, developed fever >39°C associated with rigors (CTCAE grade 2 or higher) without hemodynamic compromise 16 hours after having received 468 million Tregs (4.5 million cells/kg). The patient developed transient neutropenia, lymphopenia, and mild liver graft dysfunction (Figure 4A). Results of detailed radiological and microbiological evaluations were negative. Serum cytokine analysis revealed a significant increase in interleukin (IL)‐12 p40 (IL‐2p40), IL‐27, C‐X‐C motif chemokine ligand (CXCL)‐10 (CXCL10), C‐C motif chemokine ligand (CCL)‐2 (CCL2), IL‐5, IL‐2, interferon gamma (IFNγ), CXCL9, and CXCL11 1 day after Treg infusion, with gradual decrease by day 3 and complete normalization by day 7 (Figure 4B). As per the study protocol, the high‐grade pyrexia was considered a dose‐limiting toxicity and resulted in the expansion of the 3.0‐4.5 million Tregs/kg cohort to six participants. The infusion of Tregs did not result in serum cytokine changes in the remaining five patients receiving 4.5 million Tregs/kg (Figure 4B). Of note, the levels of IL‐12p40, IL‐18, IL‐27, IL‐33, CCL17, CCL3, CXCL10, CXCL9, and CXCL11 were already higher in P04 than in the remaining participants immediately before Treg infusion, suggesting that the adverse event may not be solely attributable to the Treg infusion.

### 3.5 Impact of Treg infusion on donor‐specific T cell responses

**Figure 5:**
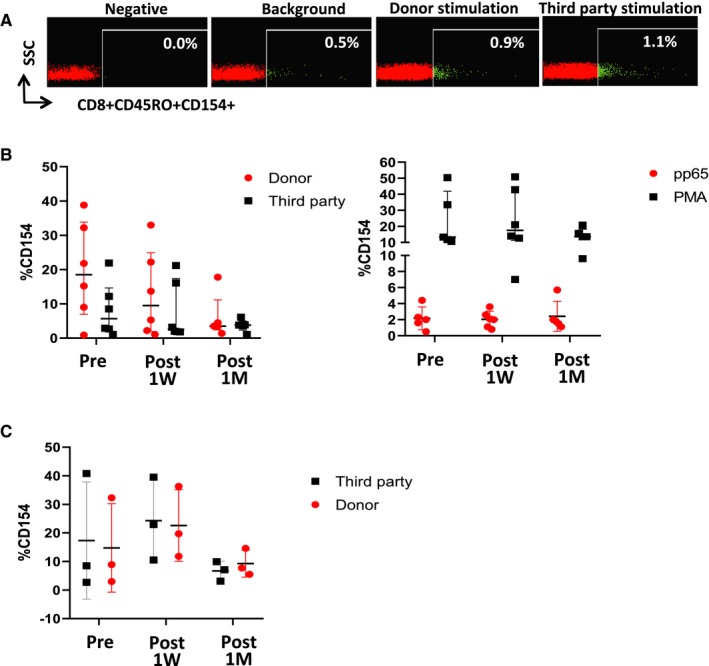
Sequential changes in donor and third‐party alloimmune responses. A, Representative dot plots corresponding to P07, displaying the expression of CD154 on memory CD8^+^ T cells collected before Treg infusion and cultured with surrogate donor or third‐party cells. B, Sequential allospecific (left panel) and CMV‐specific (right panel) memory CD8^+^ T cell responses in the six patients receiving 4.5 million Tregs/kg. C, Sequential allospecific memory CD8^+^ T cell responses in the three patients receiving 1 million Tregs/kg. For all experiments, dot plots display median and standard deviation of the proportion of CD45RO^+^ CD8^+^ T cells expressing CD154 in response to surrogate donor or third‐party cells, CMV pp65, or phorbol 12‐myristate 13‐acetate (PMA), as described [Color figure can be viewed at https://www.wileyonlinelibrary.com]

In the six recipients who received 4.5 million Tregs/kg, a gradual decrease of T cell responses (as assessed by the upregulation of CD154 on memory CD8^+^ T cells) directed against donor‐type cells was observed (*P* = .066). Although these changes did not reach statistical significance, the trend was clearly different from the responses directed against third‐party cells (*P* = .3) or the cytomegalovirus (CMV) pp65 antigen (*P* = .5), which remained stable throughout the study period. In contrast, in the three recipients dosed with 1 million Tregs/kg we observed no decrease in donor‐specific T cell responses in association with cell infusion (Figure 5).

## 4 DISCUSSION

Liver transplantation constitutes an optimal clinical scenario to explore the effects of novel immunotherapeutic approaches, as it provides an experimental setting in which the timing and identity of the antigenic challenge are known and the therapeutic intervention can be planned so as to minimize the influence of clinical confounders. Furthermore, the accumulated clinical experience with trials of immunosuppression withdrawal has provided a clear understanding of the kinetics of rejection and/or tolerance and allowed the stratification of patients according to their immunologic risk.

Our study was designed to investigate the feasibility of Treg adoptive transfer in liver transplant recipients and to determine the safety and immunologic effects of this intervention. Key aspects of the trial design were the following: (1) the isolation of Tregs immediately before transplantation; (2) the use of thymoglobulin to induce lymphodepletion and reduce effector T cells; (3) the administration of combined immunosuppression with low‐dose tacrolimus and rapamycin to minimize the deleterious effects of these drugs on Treg function^21^, ^22^; and (d) the decision to defer cell infusion until 3 months after transplant to protect Tregs from the effects of thymoglobulin and to avoid the complications often observed shortly after surgery. The overall applicability of this protocol was very low. This was due mainly to the small proportion of patients awaiting transplantation who met the strict inclusion/exclusion criteria. Additional hurdles were the unpredictable timing of the surgical procedure when employing cadaveric donors, which put considerable strain on the GMP facility; the frequent use of marginal liver grafts in our center; the development of complications either before or after transplantation that compromised the safety of the study; and the difficulties of growing large numbers of Tregs under strict GMP conditions from peripheral blood collected at the time of transplantation. As such, our experience differs greatly from the clinical study reported by Todo et al in Japan,^11^ in which 10 consecutive living donor liver transplant recipients were treated with a non‐GMP cell product. The clinical implementation of the study drastically improved after allowing inclusion of stable recipients 6‐12 months after transplant (although due to the strict eligibility criteria its overall applicability was still low). This provided enough time to perform an elective leukapheresis and reduced the high dropout rate observed when approaching patients before transplantation. Thus, following the amendment, all six participants consented were successfully dosed. Of note, thymoglobulin induction was removed from the amended protocol, as this medication has been associated with a high incidence of immune side effects such as cytokine storm when administered to patients who have not been pretreated with high‐dose immunosuppressants.^23^, ^24^

The safety profile of the Treg manufactured product was very good, with no increased incidence of infections or cancer and only a single patient experiencing an infusion reaction, classed as a dose‐limiting toxicity. Although the doses of Tregs infused were lower than what has been administered in type 1 diabetes,^7^ our highest dose was in the range of the number of Tregs contained within the cell product infused in the liver transplant trial from Japan (31‐466 × 10^6^ CD4^+^ Foxp3^+^ T cells).^11^ The lack of signs of over‐immunosuppression is very reassuring, considering that the Tregs had been expanded under polyclonal conditions and were therefore potentially capable of exerting nonspecific suppressive effects. This finding is important and has implications for the development of alternative immunotherapies currently under evaluation, such as donor‐reactive Tregs and chimeric antigen receptor (CAR)–expressing Tregs,^3^, ^25^ which preferentially recognize the transplanted organ and therefore should be even safer than the cell product tested in our trial.

Tregs are long‐lived and tend to migrate to the sites of inflammation. The kinetics of persistence and migration of ex vivo expanded Tregs following adoptive transfer, however, is still not well understood. In murine transplant models Tregs tend to accumulate in the graft and draining lymph nodes,^26^, ^27^ although Lee et al were not able to detect transferred Tregs beyond 14 days after infusion into murine islet transplant recipients.^28^ In nontransplanted nonhuman primates, adoptively transferred Tregs were shown to be short‐lived, as their numbers declined rapidly during the first week after infusion,^29^, ^30^ albeit a small number of cells were still detected both in blood and in secondary lymphoid tissues for >50 days.^29^ In human hematopoietic stem cell transplantation, infused Tregs could be tracked in blood using HLA markers up to 14 days.^5^ On the other hand, in type 1 diabetes and kidney transplant patients, expanded Tregs labeled with deuterium exhibited a peak in the circulation 7‐14 days after infusion and rapidly decreased thereafter, with approximately 20% of them being still detectable in blood 1 year after infusion.^7^, ^8^ In the patients enrolled in our study and treated with 4.5 million Tregs/kg, the number of circulating Tregs rapidly increased following infusion and remained higher than before infusion for at least 1 month. These transient changes likely corresponded to the detection of adoptively transferred Tregs, given that they were not observed after initiating rapamycin treatment and closely matched the kinetics that have been observed following the transfer of deuterium‐labeled Tregs. Furthermore, CyTOF experiments revealed that the rise in circulating Tregs was associated with increases in the specific Treg subpopulations that most closely resembled the infused Tregs. The changes in the repertoire of circulating Treg subpopulations were, however, very transient and did not persist as long as the increase in the total number of circulating CD25highCD127^−^ Tregs. This suggests that ex vivo expanded Tregs rapidly change their phenotype following infusion or, alternatively, that endogenous Tregs proliferate and contribute to the enlarged Treg compartment observed between weeks 1 and 4 postinfusion. Neither our study nor previously published reports, however, can adequately address the homing and long‐term viability of adoptively transferred Tregs. The fact that they do not persist in large numbers in the circulation may denote accelerated cell death as a result of low IL‐2 availability or preferential migration into peripheral tissues. This will remain an open question until noninvasive imaging technologies capable of tracking injected cells for long periods are successfully developed in humans.

The development of donor‐specific hyporesponsiveness is considered one of the hallmarks of transplantation tolerance. An intriguing finding of our study is the impact of the transferred Tregs on donor‐reactive T cell responses, which, in the six patients who received 4.5 million Tregs/kg, decreased 1 week after infusion and remained low 4 weeks after adoptive transfer, without obvious changes being observed in T cell responses directed against third‐party alloantigens or CMV. This is a highly unusual finding, which has not been reported in comparably stable liver, intestine, or hepatocyte transplant recipients longitudinally monitored with the same alloreactivity assay.^31^, ^32^, ^33^ The fact that patients who received 1 million/kg Tregs did not develop donor‐specific hyporesponsiveness further suggests a potential causal and dose‐effect relationship, although we cannot exclude an influence of Thymoglobulin‐induced lymphopenia, which only occurred  in the low‐dose Treg cohort. Of note, the pattern of donor‐specific T cell responses observed by Todo et al following cell infusion was similar to what we detected in our trial.^11^ Although it is not possible to formally establish a causal link between the development of donor hyporesponsiveness and Treg infusion, our findings could be explained by the preferential survival and/or proliferation after infusion of Treg clones with anti‐donor alloreactivity, which is an observation that has been documented in experimental animal models.^26^ This would be in keeping with the lack of clinically apparent nonspecific immunosuppressive effects observed following Treg infusion. Alternatively, the infused Tregs could have amplified the well‐documented capacity of liver allografts to delete donor‐reactive T cell clones.^34^, ^35^

In summary, we have described here the successful expansion under GMP conditions of polyclonal Tregs isolated from both end‐stage liver disease patients awaiting liver transplantation and stable liver transplant recipients under maintenance immunosuppression. Treg infusion was safe, well‐tolerated, and exerted a potentially beneficial effect on donor‐specific immune responses. The implementation of the clinical protocol was challenging, however, and its applicability was reliant on deferring patient recruitment and cell infusion until at least 6 months after transplant. Future studies should address the capacity of this strategy, alone or in combination with lymphodepletive therapies, to facilitate the reduction or even the complete discontinuation of antirejection medications following liver transplantation.

## DISCLOSURE

The authors of this manuscript have no conflict to disclose as described by the *American Journal of Transplantation*.

## Data Availability

The data supporting the results in the paper will be archived in an appropriate public repository.
