# Supplementary material for: Applicability, safety, and biological activity of regulatory T cell therapy in liver transplantation
Source: Am J Transplant. 2020 Feb 3;20(4):1125–36. doi: 10.1111/ajt.15700 (PMC7154724; doi:10.1111/ajt.15700)
Supplement: Supplementary file 1 [file AJT-20-1125-s001.docx]

**SUPPLEMENTARY INFORMATION**

**Supplementary Methods**

*Polyclonal Treg manufacture protocol*

At the end of the manufacture process a 2ml vial containing the individual specified dose for each patient was produced and cryopreserved in liquid nitrogen. The volume of whole blood was reduced using a Sepax 2 device (Biosafe) and Tregs were purified using the automated CliniMACS^®^ Plus system. (Miltenyi Biotec). All steps were performed in a closed system using GMP grade reagents and equipment. The enriched cells were then seeded into MACS GMP Cell expansion bags in TexMACS^TM^ GMP medium (Miltenyi Biotec) supplemented with 5% human AB serum (Seralab) and 100nM Rapamycin (Rapamune^©,^ Pfizer). The cell culture was activated with anti-CD3/CD28 coated beads (4:1 bead:cell ratio, MACS GMP ExpAct Treg Kit, Miltenyi Biotec). Human recombinant IL-2 (500 IU/ml; Proleukin^©^, Novartis) was added on day 4-6 and repeated every 2-3 days. When employing whole blood, further stimulation steps occurred on days 12 and 24, while only one additional stimulation on day 12 was required when performing leukapheresis. Fresh beads, IL-2 and Rapamycin were added at each stimulation step. At the end of the manufacturing process, which lasted 24 or 36 days depending on the number of stimulations, the following release criteria were assessed: i) CD4^+^CD25^+^FoxP3^+^ cell purity ≥60%; ii) CD8^+^ cells ≤10%; iii) potency of Treg:Teff (1:1) ≥60%; iv) no bacterial growth after a 5 day culture; v) endotoxin concentration ≤175 IU/mL; vi) no mycoplasma contamination; and vii) <100 beads per 300x10^6^cells.

*Investigational Medicinal Product (IMP) stability and GMP certification:*

Following thawing, Tregs diluted in 5% human serum albumin (HSA) had a better viability and recovery than Tregs that remained in the cryoprotectant medium (Cryostor): 87% viability and 80% recovery versus 76% and 49%, respectively. The viability of the cells diluted in 5% HAS post-thaw was maintained for 30 minutes, but dropped to 67% after 45 min. On the basis of these data the post thaw shelf life was set to 30 minutes.

*Flow cytometry immunophenotyping*: The staining protocols employed (Supplementary Table 1) were designed and standardized in collaboration with the ONE Study EU Consortium and have already been described(15). Briefly, for cell surface staining, 100 µL of anticoagulated peripheral blood was stained with extracellular antibodies for 15 min at RT in the dark, red blood cell lysed and fixed with VersaLyse +2.5% IOTest 3 fixative solution (Beckman Coulter) for 15 minutes at RT, and washed twice (PBS containing 2% FCS and 0.1% sodium azide) prior to acquisition. For the investigation of B cell subpopulations, 300 μL of anticoagulated peripheral blood was lysed with BD Pharm Lyse™ solution (BD Biosciences) for 12 minutes at RT on a tube rotator and washed twice with cold PBS. Samples were stained for 20 minutes at 4°C in the dark, fixed with 2.5% IOTest fixative solution in PBS for 15 minutes in the dark, and washed once with PBS containing 2% FCS and 0.1% sodium azide. For the investigation of intracellular markers, 300µL of anticoagulated peripheral blood was stained with extracellular antibodies, lysed and washed twice as stated above. Intracellular staining was carried out using the Foxp3/Transcription Factor Staining Buffer Set (eBioscience) according to the manufacturer’s instructions. Briefly, cells were fixed for 30 min at 4°C with the fixation/permeabilization buffer, washed and stained with intracellular antibodies in 100µL permeabilization buffer for 30 min at 4°C followed by another wash in permeabilization buffer. Flow cytometric data were collected on a Navios Flow cytometer (Beckman Coulter) and analyzed with Kaluza software (version 2.1; Beckman Coulter).

*Time-of-flight mass cytometry (CyTOF)*: We designed a panel of 39 antibodies (Supplementary Table 2) tagged with rare metal isotopes targeting both surface markers and transcription factors and employed cisplatin staining to discriminate between live and dead cells. 10^6^ events per sample were acquired on the Helios mass cytometer (Fluidigm). Data were normalized using Ce 140, Eu151, Eu153, Ho165, and Lu175 normalization beads (16). To characterize expanded and circulating Tregs we restricted the analysis to 29 out of the 39 expression markers. Heatmaps were built using Pheatmap (R package version 1.9.11) using Euclidean clustering distance and complete clustering method. To identify and visualize the different Treg subpopulations we first performed automated clustering of CD3+CD4+CD8-CD25hiFOXP3+ using t-Distributed Stochastic Neighbor Embedding (t-SNE/viSNE)(17,18). To extract the cellular hierarchy of the Treg subpopulations, we then employed Spanning-tree Progression Analysis of Density-normalized Events (SPADE)(19), setting the target number of clusters at 20. Additional T cell subpopulation analysis was performed by t-SNE on manually gated CD4+ and CD8+ cells (80000 and 45000 cells per condition respectively). To partition the cells into distinct subsets, we applied the FlowSom clustering algorithm, to identify the main immune cell sub-types in both CD4 and CD8. The number of metaclusters was manually defined to better describe the different T cell subsets. All t-SNE/viSNE, SPADE and FlowSom analyses were performed using Cytobank(20).**Supplementary Table 1: Flow Cytometry Antibody Panel**

| **Staining protocol** | **FITC / A488** | **PE** | **ECD / PE-CF594** | **PerCP.**  **Cy5.5** | **PC7** | **APC / A647** | **APCA700** | **APC-A750** | **PacBlue / BV421** | **KrOrange** |  |
| --- | --- | --- | --- | --- | --- | --- | --- | --- | --- | --- | --- |
| **1** | CD16 | CD56 | CD19 | - | CD14 | CD4 | CD8 | CD3 | CD64 | CD45 |  |
| **2** | TCRgd | TCRab | CD45RO | - | - | CD4 | CD8 | CD3 | - | CD45 |  |
| **3** | CD57 | CD28 | HLA-DR | - | CD27 | CD4 | CD8 | CD3 | CD45RA | CD45 |  |
| **4** | CD127 | CCR7 | CD62L | - | CD25 | CD4 | CD8 | CD3 | CD45RA | CD45 |  |
| **5** | IgD | CD21 | CD19 | - | CD27 | CD24 | - | CD38 | IgM | CD45 |  |
| **6** | BDCA3 | LIN | CD123 | - | CD11c | BDCA2 | - | CD16 | HLA-DR | CD45 |  |
| **7** | CD6 | CCR7 | CD62L | - | CD25 | CD4 | CD127 | CD3 | CD45RA | CD45 |  |
| **8** | CD6 | CD25 | - | - | CD4 | FOXP3 | CD127 | - | CD3 | CD45 |  |
| **9** | Vd2 | Vd1 | - | TCRgd | - | CD4 | CD8 | CD3 | TCRab | CD45 |  |
| **10** | LAG3 | CTLA4 | - | CD160 | CD4 | 2B4 | CD8 | CD3 | PD1 | CD45 |  |
| **11** | CD45RA | CD25 | GranzymeB | Helios | CD4 | FoxP3 | CD127 | CD3 | Ki67 | CD45 |  |
| **12** | CCR6 | CD25 | CD45RO | CCR4 | CD4 | CCR10 | CD127 | CD3 | CXCR3 | CD45 |  |

**Supplementary Table 2: Mass Cytometry (CyTOF) Antibody Panel**

|  |  |  |
| --- | --- | --- |
| **Marker**  **(Clone)** | | |
| CD45  (HI30) | CD152  (14D3) | CD3  (UCHT1) |
| CCR6 (G034E3) | CD27  (L128) | CD279/PD-1  (EH12.2H7) |
| CD62L  (GREG-56) | CD183  (G025H7) | Helios  (22F6) |
| CD45RA (HI100) | CXCR5  (RF8B2) | CD134/OX40 (ACT35) |
| CD19  (HIB19) | CD127  (A019D5) | CD197  G043H7 |
| CD20  (2H7) | CCR10  (314305) | CD14  (M5E2) |
| CD31  (WM59) | Gata3  (TWAJ) | CD4  (RPA-T4) |
| CD8  (RPA-T8) | Ki67  (Ki67) | CD16  (3G8) |
| CD95  (DX2) | CD25  (2A3) | Tbet  (4B10) |
| CD274/PD-L1  (29E.2A3) | CD7  (CD7-6B7) | CD184  (12G5) |
| CD45RO  (UCHL1) | FOXP3  (259D) | HLA-DR  (L243) |
| CD161  (HP-3G10) | FOXP3  (PCH101) | CD69  (FN50) |
| CD103  (Ber-ACT8) | CD38  (HIT2) | CD194/CCR4  (205410) |

**Supplementary Table 4: Flow cytometric immune phenotyping – Cohort 2**

Fresh whole blood. Subset frequency among CD45+ gated cells.


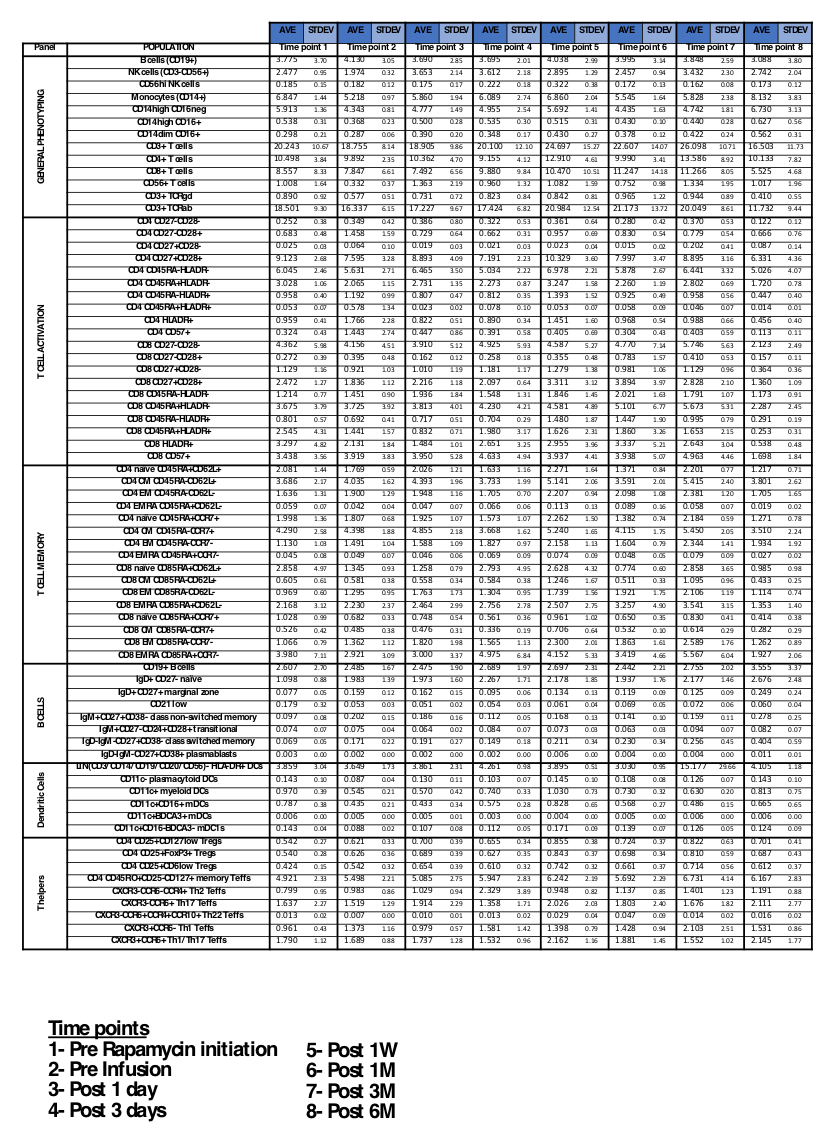


**Supplementary Table 5:** Longitudinal haematological and biochemical parameters of enrolled patients.

|  | **Pre-Treg infusion** | | | | | | | | | **Day 1 after infusion** | | | | | | | | | **Day 3 after infusion** | | | | | | | | |
| --- | --- | --- | --- | --- | --- | --- | --- | --- | --- | --- | --- | --- | --- | --- | --- | --- | --- | --- | --- | --- | --- | --- | --- | --- | --- | --- | --- |
|  | **WBC** | **Lymph** | **Plt** | **Hb** | **Bili** | **AST** | **Creat** | **Tac** | **Siro** | **WBC** | **Lymph** | **Plt** | **Hb** | **Bili** | **AST** | **Creat** | **Tac** | **Siro** | **WBC** | **Lymph** | **Plt** | **Hb** | **Bili** | **AST** | **Creat** | **Tac** | **Siro** |
| P01 | 3.65 | 0.61 | 103 | 131 | 7 | 22 | 56 | 5.1 | 6.3 | 2.95 | 0.73 | 103 | 125 | 7 | 20 | 64 | 3.5 | 6.2 | 3.73 | 0.61 | 118 | 133 | 9 | 30 | 56 | - | - |
| P02 | 2.70 | 0.41 | 186 | 108 | 6 | 21 | 148 | 4.8 | 5.2 | 2.25 | 0.37 | 170 | 96 | 5 | 20 | 140 | 3.3 | 4 | 2.48 | 0.41 | 181 | 101 | 7 | 18 | 138 | 2.8 | 3.5 |
| P03 | 4.23 | 0.41 | 287 | 99 | 4 | 11 | 133 | 4.1 | 5.1 | 3.42 | 0.34 | 256 | 94 | 4 | 12 | 122 | 3.8 | 3.7 | - | - | - | - | - | - | - | - | - |
| P04 | 5.59 | 1.65 | 278 | 120 | 6 | 28 | 74 | 4.3 | 4.5 | 4.12 | 0.51 | 175 | 113 | 9 | 31 | 75 | - | - | 3.11 | 1.19 | 186 | 110 | 9 | 62 | 72 | 2.7 | 3.5 |
| P05 | 4.51 | 1.48 | 157 | 114 | 12 | 26 | 86 | 4.1 | 3.9 | 4.37 | 1.71 | 144 | 136 | 8 | 25 | 86 | 3.9 | 4.9 | 4.46 | 1.36 | 152 | 131 | 9 | 23 | 82 | 3.3 | 4 |
| P06 | 6.26 | 1.21 | 193 | 156 | 11 | 26 | 65 | 4.5 | 5.2 | 4.79 | 1.11 | 172 | 159 | 12 | 25 | 60 | 4.9 | 4.2 | 5.80 | 1.06 | 204 | 153 | 9 | 27 | 64 | 4.7 | 3.7 |
| P07 | 3.56 | 1.41 | 212 | 127 | 12 | 26 | 60 | 4.3 | 5.3 | 3.81 | 1.61 | 208 | 132 | 14 | 27 | 64 | 3.7 | 5.1 | 4.53 | 1.36 | 220 | 123 | 5 | 28 | 55 | 2.9 | 3.8 |
| P08 | 7.64 | 1.34 | 190 | 148 | 7 | 22 | 85 | 2.8 | 2.7 | 6.88 | 1.53 | 206 | 146 | 11 | 23 | 96 | 2.5 | 2.3 | 7.55 | 1.12 | 194 | 143 | 4 | 20 | 83 | 3.4 | 2.1 |
| P09 | 5.95 | 1.11 | 315 | 107 | 5 | 20 | 100 | 5.2 | 3.6 | 4.33 | 1.00 | 300 | 110 | 8 | 20 | 100 | 4.6 | 3.5 | 4.45 | 0.99 | 300 | 107 | 5 | 19 | 98 | 4.8 | 4.2 |

|  | **Day 7 after infusion** | | | | | | | | | **1 month** | | | | | | | | | **3 months** | | | | | | | | |
| --- | --- | --- | --- | --- | --- | --- | --- | --- | --- | --- | --- | --- | --- | --- | --- | --- | --- | --- | --- | --- | --- | --- | --- | --- | --- | --- | --- |
|  | **WBC** | **Lymph** | **Plt** | **Hb** | **Bili** | **AST** | **Creat** | **Tac** | **Siro** | **WBC** | **Lymph** | **Plt** | **Hb** | **Bili** | **AST** | **Creat** | **Tac** | **Siro** | **WBC** | **Lymph** | **Plt** | **Hb** | **Bili** | **AST** | **Creat** | **Tac** | **Siro** |
| P01 | 3.92 | 0.73 | 127 | 130 | 9 | 24 | 61 | 2.9 | 5.5 | 3.62 | 0.79 | 149 | 136 | 8 | 24 | 56 | 2.6 | 5.1 | 3.18 | 0.75 | 152 | 138 | 16 | 29 | 58 | 3.3 | 8.1 |
| P02 | 2.48 | 0.39 | 203 | 104 | 8 | 10 | 135 | 4.1 | 4.2 | 2.17 | 0.53 | 228 | 119 | 6 | 21 | 141 | 4.1 | 5.7 | 2.16 | 0.30 | 247 | 118 | 7 | 25 | 130 | 3.1 | 4 |
| P03 | - | - | - | - | - | - | - | - | - | 2.50 | 0.30 | 255 | 91 | 3 | 11 | 146 | - | - | - | - | - | - | - | - | - | - | - |
| P04 | 4.21 | 2.49 | 234 | 117 | 4 | 108 | 71 | 6.6 | 4.1 | 5.84 | 2.81 | 321 | 117 | 5 | 28 | 73 | 4.8 | 4.7 | 7.71 | 3.18 | 348 | 135 | 5 | 32 | 77 | 3.8 | 4.8 |
| P05 | 4.71 | 1.72 | 195 | 130 | 7 | 29 | 78 | 2.7 | 3.6 | 5.03 | 1.38 | 165 | 136 | 10 | 46 | 82 | 3.6 | 4 | 4.94 | 1.52 | 210 | 148 | 9 | 24 | 83 | 4.9 | 4.1 |
| P06 | 6.24 | 1.08 | 212 | 157 | 10 | 30 | 60 | 3.6 | 3.3 | 6.80 | 1.22 | 220 | 154 | 10 | 24 | 64 | 3.5 | 3.8 | 7.03 | 1.16 | 255 | 154 | 7 | 21 | 49 | 2.4 | 3.8 |
| P07 | 3.49 | 1.51 | 193 | 124 | 8 | 26 | 62 | 4.6 | 4.2 | 3.29 | 1.23 | 216 | 137 | 12 | 31 | 66 | 2.8 | 4.3 | 3.61 | 1.46 | 202 | 134 | 11 | 29 | 68 | 3.5 | 4.4 |
| P08 | 5.73 | 0.97 | 173 | 148 | 5 | 20 | 78 | 2.9 | 4.4 | 6.39 | 1.19 | 193 | 153 | 3 | 25 | 78 | 3.3 | 1.8 | 5.61 | 0.95 | 163 | 153 | 4 | 37 | 83 | 3.4 | 4.2 |
| P09 | 4.85 | 0.92 | 296 | 106 | 6 | 18 | 109 | 4.2 | 4.2 | 5.55 | 0.89 | 259 | 117 | 7 | 24 | 100 | 4.6 | 3.8 | 3.50 | 0.95 | 250 | 128 | 5 | 26 | 106 | 5.2 | 4.3 |

|  | **6 months** | | | | | | | | |
| --- | --- | --- | --- | --- | --- | --- | --- | --- | --- |
|  | **WBC** | **Lymph** | **Plt** | **Hb** | **Bili** | **AST** | **Creat** | **Tac** | **Siro** |
| P01 | 5.18 | 1.03 | 163 | 144 | 16 | 26 | 72 | 2.8 | 5 |
| P02 | 1.95 | 0.63 | 251 | 128 | 10 | 36 | 145 | 4.4 | 4.5 |
| P03 | - | - | - | - | - | - | - | - | - |
| P04 | 6.99 | 2.26 | 289 | 122 | 5 | 36 | 76 | 3.2 | 3.5 |
| P05 | 3.00 | 1.02 | 175 | 144 | 10 | 33 | 88 | 3.3 | 4.1 |
| P06 | 7.31 | 1.50 | 250 | 160 | 8 | 22 | 58 | 3.4 | 4 |
| P07 | 3.15 | 1.32 | 189 | 140 | 10 | 26 | 72 | 3.9 | 4.6 |
| P08 | 6.19 | 1.17 | 164 | 154 | 6 | 30 | 85 | 4 | 5.2 |
| P09 | 6.06 | 1.08 | 278 | 131 | 5 | 19 | 92 | 5.2 | 4.6 |

Abbreviations: WBC = white blood count (10^9^/L), Lympho = lymphocyte count (10^9^/L), Plt = platelet count (10^9^/L), Hb = haemoglobin (g/L), Bili = bilirubin (μmol/L), AST = aspartate aminotransferase (IU/L), Creat = creatinine (μmol/L), Tac = tacrolimus level (μg/L), Siro = sirolimus level (μg/L).

**Supplementary Figure 1. Selected immunophenotypic markers on circulating and expanded Tregs as assessed by CyTOF.**

**Supplementary Figure 2.** (A) Frequency of circulating Tregs in the 20 nodes determined by SPADE analysis from viSNE clustering when employing the expression levels of the top 10 genes differentially expressed between the expanded Tregs and the pre-infusion circulating Tregs (Ki67, CD38, OX40, CD25, CD69, GATA3, CCR4, CTLA4, PD1, HLA-DR). (B) Median expression of top 10 differently expressed genes in the 20 nodes from circulating Tregs at Pre Infusion time point. (C) Representative SPADE clustering of circulating Tregs based on the viSNE analysis identifying the 20 nodes. Bubble size and colour determine population density.

**Supplementary Figure 3.** Blood samples from Patients at different time points were stained using the panel described in Supplementary Table 2 and acquired on the CyTOF-2 mass cytometer. CD3+CD4+ T cells were manually gated and 80000 were clustered using Flowsom algorithm. The number of metaclusters were manually defined for better describing the different CD4 subsets. (A) Representative overlaying plot showing the 25 metaclusters and their identify (B) based on the cumulative heatmap showing the expression of the different markers (C).

**Supplementary Figure 4.** Blood samples from Patients at different time points were stained using the panel described in Supplementary Table 2 and acquired on the CyTOF-2 mass cytometer. CD3+CD8+ T cells were manually gated and 45000 were clustered using Flowsom algorithm. The number of metaclusters were manually defined for better describing the different CD8 subsets. (A) Representative overlaying plot showing the 24 metaclusters and their identify (B) based on the cumulative heatmap showing the expression of the different markers (C).
